# Association of Postbreakfast Triglyceride and Visit-to-Visit Annual Variation of Fasting Plasma Glucose with Progression of Diabetic Nephropathy in Patients with Type 2 Diabetes

**DOI:** 10.1155/2016/4351376

**Published:** 2016-11-15

**Authors:** Kaori Kitaoka, Akiko Takenouchi, Ayaka Tsuboi, Keisuke Fukuo, Tsutomu Kazumi

**Affiliations:** ^1^Research Institute for Nutrition Sciences, Mukogawa Women's University, Nishinomiya, Hyogo, Japan; ^2^Department of Nutritional Sciences for Well-Being, Faculty of Health Sciences for Welfare, Kansai University of Welfare Sciences, Kashiwara, Japan; ^3^Department of Food Sciences and Nutrition, School of Human Environmental Sciences, Mukogawa Women's University, 6-46 Ikebiraki-Cho, Nishinomiya, Hyogo 663-8558, Japan; ^4^Diabetes Division, Sadamitsu Hospital, Kakogawa, Hyogo 675-0005, Japan

## Abstract

Urinary albumin/creatinine ratio (ACR) was measured at baseline and after a median follow-up of 6.0 years in 161 patients with type 2 diabetes. Intrapersonal means and SD of HbA1c, systolic BP, fasting, and postmeal plasma glucose (FPG and PMPG, resp.) and serum triglycerides (FTG and PMTG, resp.) were calculated in each patient during the first 12 months after enrollment. Associations of these variables with nephropathy progression (15 patients with progression of albuminuric stages and 5 with ACR doubling within the microalbuminuric range) were determined by multivariate logistic regression analysis providing odds ratio with 95% confidential interval. Patients with nephropathy progression, compared with those without nephropathy progression, had higher HbA1c (*p* < 0.01). They also had higher means and SD of FPG (both *p* < 0.05), FTG (both *p* < 0.05), and PMTG (*p* = 0.001). Multivariate logistic regression analysis demonstrated that SD-FPG (1.036, 1.001–1.073, *p* = 0.04) and PMTG (1.013, 1.008–1.040, *p* = 0.001) were significant predictors of progression of nephropathy even after adjustment for mean FPG and SD-FTG, age, sex, BMI, waist circumference, diabetes duration and therapy, means and SDs of HbA1c, PPG, FTG and systolic BP, baseline ACR, smoking status, and uses of antihypertensive and lipid-lowering medications. Consistency of glycemic control and management of postmeal TG may be important to prevent nephropathy progression in type 2 diabetic patients.

## 1. Introduction

Diabetes is an important cause of mortality and morbidity worldwide through both direct clinical sequelae and increased mortality from cardiovascular and kidney diseases [1]. Long-term glycemic control, as expressed by hemoglobin (Hb) A1c levels, is the main risk factor for the development of microvascular complications including diabetic kidney disease [2, 3]. Among patients with diabetes mellitus, elevated blood pressure (BP) is associated with progression of microvascular complications such as nephropathy and retinopathy [4]. In addition to high BP and hyperglycemia, dyslipidemia, characterized by high fasting triglycerides (TG) and low HDL cholesterol, has an important role in the progression of kidney disease in patients with diabetes [5]. Although a number of observational studies have reported that dyslipidemia may be associated with albuminuria, renal function impairment, and end-stage renal disease in the general population, studies are scarce investigating a relationship between dyslipidemia and albuminuria in diabetes [6]. Cross-sectional studies have found an association between fasting serum triglycerides (FTG) and the development of micro- and macroalbuminuria [7, 8]. In a large multinational case-control study of 2,535 type 2 diabetics with good control of LDL cholesterol, FTG and HDL cholesterol were associated with a higher risk of diabetic kidney disease defined as either proteinuria > 300 mg/L, albuminuria, or estimated glomerular filtration rate (eGFR) < 60 mL/min/1.73 m^2^ [9].

The majority of patients with type 2 diabetes show high and prolonged postprandial lipemia after meals [10]. It has been reported that type 2 diabetic patients with microalbuminuria have higher postprandial triglyceridemia than those without microalbuminuria after ingestion of a mixed test meal [11]. However, we are not aware of studies that examined associations of postmeal TG (PMTG) with changes in albuminuric stages despite the fact that the vasculature is commonly exposed to prolonged and exaggerated postprandial triglyceridemia, especially in type 2 diabetic patients [12].

There is emerging interest to examine the influence of glycemic and BP variance in diabetic vascular complications [13, 14]. Recently, we have shown direct association of annual HbA1c variability with kidney function decline in type 2 diabetic patients [15]. We now asked the question whether means and annual variabilities of FTG and PMTG as well as fasting and postmeal plasma glucose (FPG and PMPG, resp.), HbA1c, and BP might be associated with progression of nephropathy in patients with type 2 diabetes.

## 2. Subjects and Methods

We here show results of 161 patients, in whom urinary albumin/creatinine ratio (ACR) was measured at baseline and after a follow-up, out of 168 patients with type 2 diabetes whose details have been reported elsewhere [15]. After the first visit in 2005 they were followed up in the subsequent at least 24 months through December 31, 2012, to assess urinary ACR with a median follow-up of 6.0 years (interquartile range; 4.1–6.5 years). Study protocol was consistent with the Japanese Government's Ethical Guidelines Regarding Epidemiological Studies in accordance with the Declaration of Helsinki.

For each subject on each monthly visit, BP was measured and blood was withdrawn at 2 h after breakfast taken at home and after an overnight fasting as previously reported in details [15]. Plasma glucose, serum lipids and lipoproteins, creatinine, uric acid, and other blood tests were measured by standard methods using an autoanalyzer. HbA1c values were determined by high performance liquid chromatography. Intrapersonal means and SD of systolic BP, HbA1c, FPG, PMPG, FTG, and PMTG obtained during the first 12 months after enrollment were calculated as previously reported [15].

Urinary albumin was measured in random urine samples using a turbidimetric immunoassay and expressed as ACR. Normoalbuminuria, microalbuminuria, and macroalbuminuria were defined as an ACR < 30 mg/g, ACR between 30 and 299 mg/g, and ACR ≧ 300 mg/g, respectively [16]. Progression of nephropathy was defined as the progression of albuminuric stages and doubling of ACR within the microalbuminuric range. Serum and urinary creatinine were measured enzymatically and eGFR was determined using the equation recommended by the Japanese Society for Nephrology [17]. Logistic regression was used to estimate odds of nephropathy progression over 6 years in relation to baseline variables.

Data were presented as mean ± SE unless otherwise stated. Differences between 2 groups were analyzed by *t* test and frequencies of conditions by Chi-square tests. Bivariate and multiple logistic regression analysis were performed. Results were expressed as odd ratios (OR) with their 95% confidence intervals (CI). A two-tailed *p* < 0.05 was considered statistically significant. All calculations were performed with SPSS system 15.0 (SPSS Inc., Chicago, IL).

## 3. Results

As previously reported [15], patients had relatively good glycemic, lipid, and BP control (Table 1). Among 161 patients, 109, 45, and 7 patients had normoalbuminuria, microalbuminuria, and macroalbuminuria at baseline, respectively. There was no difference in clinical features between 109 and 7 normoalbuminuric patients who were excluded from the analysis (data not shown). Progression of albuminuric stages occurred in 15 patients (14 patients with normoalbuminuria to microalbuminuria and 1 to macroalbuminuria) and doubling of ACR in 5 patients: 20 patients (12.4%) had progression of nephropathy and they are termed as progressors. Remission of albuminuric stages and ACR reduction by 50% within the microalbuminuric range occurred in 22 and 6 patients, respectively; 28 patients (17.4%) had remission of nephropathy.

Progressors had higher HbA1c and mean and SD of FPG than nonprogressors (Table 1), whereas there was no difference in SD-HbA1c and mean and SD of PMPG between the 2 groups. However, duration and treatment of diabetes, anthropometric variables, and proportion of male patients did not differ. Progressors also had higher mean and SD of not only FTG but PMTG while HDL and LDL cholesterol did not differ between the 2 groups. Although baseline ACR concentrations did not differ between the 2 groups, microalbuminuria tended to be more prevalent in patients with compared with those without nephropathy progression. There was no difference in mean and SD of systolic BP, percentages of users of inhibitors of renin-angiotensin system, uric acid concentrations, and eGFR.

Bivariate logistic regression analysis was done in variables which showed significant difference between progressors and nonprogressors (Table 2). HbA1c and mean and SD of FPG were associated with progression of nephropathy. Further, mean and SD of FTG and PMTG were associated with nephropathy progression. Multiple logistic regression analysis (Table 3) has demonstrated that PMTG and SD of FPG were associated with progression of nephropathy independently of SD of PMTG, mean FPG, age, sex, BMI, waist circumference, diabetes duration and therapy, means and SDs of HbA1c, FTG and systolic BP, baseline ACR, smoking status, and uses of antihypertensive and lipid-lowering medications.

## 4. Discussion

To the best of our knowledge, the present study is the first to demonstrate associations of progression of nephropathy with annual mean and visit-to-visit variability of FPG as well as mean HbA1c in type 2 diabetes patients. In addition, nephropathy progression was associated with annual mean and visit-to-visit variability of FTG. Further, it was associated with annual mean and visit-to-visit variability of PMTG. Among those glycemic and lipid variables, postmeal TG and visit-to-visit variability of FPG were predictors of progression of nephropathy in type 2 diabetes patients with preserved kidney function and relatively good glycemic, lipid, and BP control at baseline independently of mean FPG, HbA1c, FTG, and known predictors of albuminuria progression [18].

Recent experimental studies have demonstrated a direct effect of saturated fatty acids on podocyte function and thus a potential link between high TG, systemic or organ-specific insulin resistance, and the development of albuminuria [19]. The role of hypertriglyceridemia in progression of renal disease is also supported by data that treatment with fenofibrate decreased ACR concentrations [20] and the progression to microalbuminuria [21]. High FTG levels predicted a risk of developing proteinuria in a large screened cohort in a retrospective and longitudinal Japanese study [22]. FTG were shown to predict prospectively a risk of albuminuria progression in patients with diabetes as well [21, 23, 24]. However, we are not aware of previous studies investigating whether postprandial triglyceridemia might be associated with progression of albuminuria. The present study is the first to demonstrate that PMTG is a predictor of albuminuria progression in type 2 diabetic patients independently of FTG and known predictors of albuminuria progression [18]. In our study, eGFR at baseline averaged 76 ± 16 mL/min/1.73 m^2^ and only 16% of patients had eGFR < 60 mL/min/1.73 m^2^ [15], and it is thus unlikely that the elevation of triglycerides at baseline could be explained by renal dysfunction per se [25].

Long-term instability of FPG was a predictor of cardiovascular and all-cause mortality in the Verona Diabetes Study [26–29]. Annual variation of FPG also was a predictor of ischemic stroke, cancer incidence and mortality in the Taichung Diabetes Study [30–32]. In a 5-year follow-up prospective cohort study [33], FPG variability was a predictor of the development of retinopathy in type 2 diabetic patients whereas the Verona Diabetes Study did not find this association [34]. To our knowledge, the current study is the first to demonstrate an association of annual variation in FPG with the progression of nephropathy. Several mechanisms including oxidative stress and inflammation may be involved in the association between glycemic variability and microvascular complications in diabetes as recently discussed in detail [35].

The strength of the current study is that means and variabilities of fasting and postprandial PG and TG were calculated using 6 measurements obtained during a 1-year period in the majority of participants. Postmeal TG was measured after breakfast eaten at home: in real-life conditions. In addition, mean and SD of HbA1c were calculated using 12 measurements in the current study. Such a testing frequency is routine in clinical settings in Japan. Frequent measurement could contribute to the reliability of data. Finally, means and variability of BP also have been taken into accounted. Major limitation is that ACR was measured once each at the entry and at the end of the study. Other limitations are that study participants were small in number and from a single clinic in Japan. However, the characteristics of our study participants are similar to those reported in a previous large-scale study in Japan [36].

## 5. Conclusions

The current study has demonstrated that postmeal TG and visit-to-visit variability of FPG were significant predictors of progression of nephropathy in type 2 diabetic patients. These findings suggest that consistency of glycemic control and management of postmeal TG may be important to prevent nephropathy progression in type 2 diabetic patients. Further studies are needed to confirm the association in other ethnic groups with more patients.

## Figures and Tables

**Table 1:** Characteristics of patients with progression of diabetic nephropathy.

	Progression of nephropathy		*p* values
	No (*n* = 141)	Yes (*n* = 20)	
Male sex (*n*, %)	67, 47.5	8, 40.0	0.528
Age (years)	62.6 ± 0.8	62.5 ± 2.2	0.951
BMI (kg/m^2^)	23.9 ± 0.3	25.0 ± 0.04	0.206
Waist circumference (cm)	86.5 ± 0.9	88.2 ± 1.9	0.464
Duration of diabetes (years)	9.6 ± 0.6	12.2 ± 1.9	0.158
Treatment of diabetes; diet/OHA/insulin (%)	32.6/51.1/16.3	25.0/50.0/25.0	0.766
Hypertension; CCB/RASi/diuretics (%)	34.8/44.3/4.3	45.0/30.0/10.0	0.357
FPG (mg/dL)	124 ± 1.7	134 ± 0.01	0.037
SD-FPG (mg/dL)	17.1 ± 1.1	23.7 ± 4.1	0.040
PMPG (mg/dL)	151 ± 4.0	167 ± 14.4	0.172
SD-PMPG (mg/dL)	33.2 ± 1.7	37.2 ± 4.6	0.424
HbA1c (%)	6.93 ± 0.06	7.46 ± 0.24	0.004
SD-HbA1c (%)	0.45 ± 0.04	0.62 ± 0.11	0.116
Total cholesterol (mg/dL)	188 ± 1.7	191 ± 5.7	0.489
HDL cholesterol (mg/dL)	56 ± 1.3	53 ± 3.7	0.499
LDL cholesterol (mg/dL)	112 ± 1.7	112 ± 6.8	0.995
FTG (mg/dL)	111 ± 3.9	145 ± 16.8	0.005
SD-FTG (mg/dL)	29.4 ± 1.7	40.2 ± 6.0	0.037
PMTG (mg/dL)	140 ± 5.2	191 ± 19.8	0.001
SD-PMTG (mg/dL)	35.8 ± 1.9	54.4 ± 7.2	0.001
Urinary ACR (mg/g)	92 ± 29.4	43 ± 9.8	0.531
Microalbuminuria (*n*, %)	36, 25.5	9, 45.0	0.069
Serum creatinine (mg/dL)	0.75 ± 0.01	0.81 ± 0.08	0.156
eGFR (mL/min/1.73 m^2^)	75.7 ± 1.3	75.6 ± 4.7	0.971
eGFR < 60 mL/min/1.73 m^2^ (*n*, %)	23, 16.3	2, 10.0	0.466
Uric acid (mg/dL)	5.1 ± 0.1	5.6±0.4	0.149
Systolic BP (mmHg)	128 ± 1.0	131 ± 3.2	0.344
SD-systolic BP (mmHg)	10.3 ± 0.3	10.2 ± 0.5	0.934
Diastolic BP (mmHg)	72 ± 0.6	72 ± 1.8	0.816

Mean ± SE unless otherwise stated. OHA: oral hypoglycemic agents, CCB: calcium channel blockers, RASi: renin-angiotensin system inhibitors, PG: plasma glucose, SD: standard deviation, eGFR: estimated glomerular filtration rate, BP: blood pressure, ACR: albumin/creatinine ratio.

**Table 2:** Bivariate logistic regression analysis for progression of nephropathy.

	OR	95% CI		*p* values
		Lower	Upper	
FPG	1.02	1.001	1.041	0.042
SD-FPG	1.03	1.000	1.058	0.049
HbA1c	2.25	1.261	4.017	0.006
FTG	1.01	1.002	1.018	0.011
SD-FTG	1.02	1.000	1.039	0.045
PMTG	1.01	1.004	1.018	0.003
SD-PMTG	1.03	1.010	1.052	0.003

OR: odds ratio, CI: confidential interval. Other abbreviations are the same as in Table 1.

**Table 3:** Multivariate logistic regression analysis for progression of nephropathy.

	OR	95% CI		*p* values
		Lower	Upper	
SD-FPG	1.04	1.0002	1.0725	0.049
PMTG	1.01	1.0053	1.0213	0.001

Other independent variables included the following: age, sex, BMI, waist circumference, duration and treatment of diabetes, log ACR and systolic blood pressure, smoking status, and uses of antihypertensive and lipid-lowering medications. OR: odds ratio, CI: confidential interval. Other abbreviations are the same as in Table 1.

## References

[1] Danaei G., Lawes C. M., Vander Hoorn S., Murray C. J., Ezzati M. (2006). Global and regional mortality from ischaemic heart disease and stroke attributable to higher-than-optimum blood glucose concentration: comparative risk assessment. *The Lancet*.

[2] Lachin J. M., Genuth S., Nathan D. M., Zinman B., Rutledge B. N. (2008). Effect of glycemic exposure on the risk of microvascular complications in the diabetes control and complications trial—revisited. *Diabetes*.

[3] Stratton I. M., Adler A. I., Neil H. A. W. (2000). Association of glycaemia with macrovascular and microvascular complications of type 2 diabetes (UKPDS 35): prospective observational study. *British Medical Journal*.

[4] Adler A. I., Stratton I. M., Neil H. A. W. (2000). Association of systolic blood pressure with macrovascular and microvascular complications of type 2 diabetes (UKPDS 36): prospective observational study. *British Medical Journal*.

[5] Rutledge J. C., Ng K. F., Aung H. H., Wilson D. W. (2010). Role of triglyceride-rich lipoproteins in diabetic nephropathy. *Nature Reviews Nephrology*.

[6] Hung C.-C., Tsai J.-C., Kuo H.-T., Chang J.-M., Hwang S.-J., Chen H.-C. (2013). Dyslipoproteinemia and impairment of renal function in diabetic kidney disease: an analysis of animal studies, observational studies, and clinical trials. *Review of Diabetic Studies*.

[7] Samuelsson O., Mulec H., Knight-Gibson C. (1997). Lipoprotein abnormalities are associated with increased rate of progression of human chronic renal insufficiency. *Nephrology Dialysis Transplantation*.

[8] Penno G., Solini A., Zoppini G. (2015). Hypertriglyceridemia is independently associated with renal, but not retinal complications in subjects with type 2 diabetes: a cross-sectional analysis of the Renal Insufficiency and Cardiovascular Events (RIACE) Italian Multicenter Study. *PLoS ONE*.

[9] Sacks F. M., Hermans M. P., Fioretto P. (2014). Association between plasma triglycerides and high-density lipoprotein cholesterol and microvascular kidney disease and retinopathy in type 2 diabetes mellitus: a global case-control study in 13 countries. *Circulation*.

[10] Ginsberg H. N., Zhang Y.-L., Hernandez-Ono A. (2005). Regulation of plasma triglycerides in insulin resistance and diabetes. *Archives of Medical Research*.

[11] Tentolouris N., Stylianou A., Lourida E. (2007). High postprandial triglyceridemia in patients with type 2 diabetes and microalbuminuria. *Journal of Lipid Research*.

[12] Pastromas S., Terzi A.-B., Tousoulis D., Koulouris S. (2008). Postprandial lipemia: an under-recognized atherogenic factor in patients with diabetes mellitus. *International Journal of Cardiology*.

[13] Gorst C., Kwok C. S., Aslam S. (2015). Long-term glycemic variability and risk of adverse outcomes: a systematic review and meta-analysis. *Diabetes Care*.

[14] Parati G., Liu X., Ochoa J. E. (2014). Clinical relevance of visit-to-visit blood pressure variability: impact on renal outcomes. *Journal of Human Hypertension*.

[15] Takenouchi A., Tsuboi A., Terazawa-Watanabe M., Kurata M., Fukuo K., Kazumi T. (2015). Direct association of visit-to-visit HbA1c variation with annual decline in estimated glomerular filtration rate in patients with type 2 diabetes. *Journal of Diabetes and Metabolic Disorders*.

[16] American Diabetes Association (2015). Microvascular complications and foot care. *Diabetes Care*.

[17] Matsuo S., Imai E., Horio M. (2009). Revised equations for estimated GFR from serum creatinine in Japan. *American Journal of Kidney Diseases*.

[18] Afghahi H., Cederholm J., Eliasson B. (2011). Risk factors for the development of albuminuria and renal impairment in type 2 diabetes—the Swedish National Diabetes Register (NDR). *Nephrology Dialysis Transplantation*.

[19] Lennon R., Pons D., Sabin M. A. (2009). Saturated fatty acids induce insulin resistance in human podocytes: implications for diabetic nephropathy. *Nephrology Dialysis Transplantation*.

[20] Kazumi T., Hirano T., Yoshino G. (2003). Effects of fenofibrate on albuminuria in patients with hypertriglyceridemia and/or hyperuricemia: a multicenter, randomized, double-blind, placebo-controlled, crossover study. *Current Therapeutic Research—Clinical and Experimental*.

[21] Keech A., Simes R. J., Barter P. (2005). Effects of long-term fenofibrate therapy on cardiovascular events in 9795 people with type 2 diabetes mellitus (the FIELD study): randomised controlled trial. *The Lancet*.

[22] Tozawa M., Iseki K., Iseki C., Oshiro S., Ikemiya Y., Takishita S. (2002). Triglyceride, but not total cholesterol or low-density lipoprotein cholesterol levels, predict development of proteinuria. *Kidney International*.

[23] Retnakaran R., Cull C. A., Thorne K. I., Adler A. I., Holman R. R., UKPDS Study Group (2006). Risk factors for renal dysfunction in type 2 diabetes: U.K. Prospective Diabetes Study 74. *Diabetes*.

[24] Hadjadj S., Duly-Bouhanick B., Bekherraz A. (2004). Serum triglycerides are a predictive factor for the development and the progression of renal and retinal complications in patients with type 1 diabetes. *Diabetes and Metabolism*.

[25] Attman P.-O., Samuelsson O. (2009). Dyslipidemia of kidney disease. *Current Opinion in Lipidology*.

[26] Muggeo M., Verlato G., Bonora E. (1995). Long-term instability of fasting plasma glucose predicts mortality in elderly NIDDM patients: the Verona Diabetes Study. *Diabetologia*.

[27] Muggeo M., Verlato G., Bonora E., Zoppini G., Corbellini M., de Marco R. (1997). Long-term instability of fasting plasma glucose, a novel predictor of cardiovascular mortality in elderly patients with non-insulin-dependent diabetes mellitus: the Verona Diabetes Study. *Circulation*.

[28] Muggeo M., Zoppini G. E., Brun E., Bonadonna R. C., Moghetti P., Verlato G. (2000). Fasting plasma glucose variability predicts 10-year survival of type 2 diabetic patients: the Verona Diabetes Study. *Diabetes Care*.

[29] Zoppini G., Verlato G., Targher G., Bonora E., Trombetta M., Muggeo M. (2008). Variability of body weight, pulse pressure and glycaemia strongly predict total mortality in elderly type 2 diabetic patients. The Verona Diabetes Study. *Diabetes/Metabolism Research and Reviews*.

[30] Lin C.-C., Li C.-I., Liu C.-S. (2012). Annual fasting plasma glucose variation increases risk of cancer incidence and mortality in patients with type 2 diabetes: the Taichung Diabetes Study. *Endocrine-Related Cancer*.

[31] Lin C.-C., Li C.-I., Yang S.-Y. (2012). Variation of fasting plasma glucose: a predictor of mortality in patients with type 2 diabetes. *The American Journal of Medicine*.

[32] Lin C.-C., Yang C.-P., Li C.-I. (2014). Visit-to-visit variability of fasting plasma glucose as predictor of ischemic stroke: competing risk analysis in a national cohort of Taiwan Diabetes Study. *BMC Medicine*.

[33] Gimeno-Orna J. A., Castro-Alonso F. J., Boned-Juliani B., Lou-Arnal L. M. (2003). Fasting plasma glucose variability as a risk factor of retinopathy in Type 2 diabetic patients. *Journal of Diabetes and Its Complications*.

[34] Zoppini G., Verlato G., Targher G. (2009). Is fasting glucose variability a risk factor for retinopathy in people with type 2 diabetes?. *Nutrition, Metabolism and Cardiovascular Diseases*.

[35] Cavalot F. (2013). Do data in the literature indicate that glycaemic variability is a clinical problem? Glycaemic variability and vascular complications of diabetes. *Diabetes, Obesity and Metabolism. Supplement*.

[36] Sone H., Tanaka S., Iimuro S. (2010). Long-term lifestyle intervention lowers the incidence of stroke in Japanese patients with type 2 diabetes: a nationwide multicentre randomised controlled trial (the Japan Diabetes Complications Study). *Diabetologia*.

